# The Development and Application of Bispecific Antibodies in B-Cell Non-Hodgkin Lymphoma

**DOI:** 10.3390/jpm15020051

**Published:** 2025-01-28

**Authors:** Laura Sun, Jason T. Romancik

**Affiliations:** 1 Department of Pharmacy, Winship Cancer Institute of Emory University, Atlanta, GA 30308, USA; laura.sun@emoryhealthcare.org; 2 Department of Hematology and Medical Oncology, Winship Cancer Institute of Emory University, Atlanta, GA 30308, USA

**Keywords:** B-cell non-Hodgkin lymphoma, bispecific antibody, targeted therapy, immunotherapy

## Abstract

T-cell-engaging bispecific antibodies (BsAbs) are monoclonal antibodies that redirect the cytotoxic activity of T-cells to target malignant neoplasms. B-cell non-Hodgkin lymphoma (B-NHL) is a heterogenous group of aggressive and indolent malignancies with significant therapeutic challenges due to high relapse rates and limited options for relapsed/refractory disease. BsAbs function by simultaneously binding to CD3 on endogenous T-cells and a tumor-associated antigen, creating an immunologic synapse which results in the death of the target cell. The widespread T-cell activation that occurs with BsAb administration can result in cytokine release syndrome and neurological adverse events. Mosunetuzumab, epcoritamab, and glofitamab are CD20-targeting BsAbs that have demonstrated promising single-agent activity in both indolent and aggressive B-NHL. BsAbs are now being evaluated in combination with other anti-lymphoma agents and in earlier lines of treatment, and the results of ongoing clinical trials involving these agents have the potential to reshape the treatment landscape for B-NHL. In this review, we describe the structural features, clinical data, and toxicity profile associated with the BsAbs currently used to treat B-NHL and then discuss ongoing studies and future directions for this exciting new class of therapeutic agents.

## 1. Introduction

B-cell non-Hodgkin lymphoma (B-NHL) refers to a heterogeneous group of malignant neoplasms that can be broadly categorized into aggressive and indolent subtypes. Aggressive subtypes of B-NHL, such as diffuse large-B-cell lymphoma (DLBCL) and high-grade B-cell lymphoma (HGBCL), are treated with curative intent using multiagent chemoimmunotherapy (CIT) as the initial treatment [1]. In contrast, indolent subtypes of B-NHL, such as follicular lymphoma (FL), are generally considered incurable at advanced stages, although current treatments can induce prolonged remission and many patients live for decades [2]. However, many patients with B-NHL relapse and have poor survival even in the modern era, so there remains a need for more effective therapies.

Developing immunotherapies that redirect and enhance the anti-tumor functions of the immune system has long been a goal of cancer research, and we are fortunate to now have several such agents available to treat B-NHL. Rituximab, an anti-CD20 monoclonal antibody (mAb), was the first antibody-based therapy to receive regulatory approval in oncology, and its use has significantly improved outcomes for patients with B-NHL [3,4]. More recently, the development of chimeric antigen receptor T-cell therapy (CAR-T) represented another major milestone in cancer immunotherapy. CAR-T involves collecting T-cells from a patient and genetically modifying them ex vivo to target tumor antigens and trigger the cytotoxic death of the malignant cells upon their return to the patient. CAR-T results in prolonged remission and is a potential cure for some patients with relapsed or refractory (R/R) B-NHL, but preventing CAR-T failure, mitigating treatment toxicity, and improving access to care remain significant challenges associated with this treatment modality [5,6,7,8,9].

T-cell-engaging bispecific antibodies (BsAbs) have emerged as one of the most promising new forms of immunotherapy in the field of oncology. BsAbs simultaneously target a T-cell antigen and a tumor-associated antigen to redirect the cytotoxic activity of T-cells toward the tumor. For B-NHL, the BsAbs mosunetuzumab, epcoritamab, and glofitamab are currently approved by the US Food and Drug Administration (FDA) to treat patients with relapsed disease. All three of these agents co-target CD20 and CD3 (CD20×CD3) and have demonstrated encouraging single-agent activity in phase 2 studies [10,11,12,13]. Rapid T-cell activation and the secretion of supraphysiologic levels of inflammatory cytokines cause cytokine release syndrome (CRS) and immune effector cell-associated neurotoxicity syndrome (ICANS), which are two of the major toxicities associated with these agents. BsAbs have been developed for use in B-NHL, and there are now commercially available agents to treat patients with R/R DLBCL and R/R FL. Several ongoing clinical trials evaluating BsAb-containing combinations in earlier lines of treatment are currently underway; results from these studies may soon reshape the treatment landscape for B-NHL.

This review explores the development of BsAbs, with a focus on describing their structural and pharmacokinetic properties, reviewing their current role in treating B-NHL, and discussing future directions for this exciting class of agents.

## 2. Early Development of Bispecific Antibodies for B-NHL

Anti-CD20 mAbs such as rituximab and obinutuzumab activate the innate immune system via their fragment crystallizable (Fc) receptors, resulting in antibody-dependent cellular cytotoxicity (ADCC), antibody-dependent cellular phagocytosis (ADCP), and complement-dependent cytotoxicity (CDC) of the target cells [14]. However, these mAbs do not recruit cytotoxic T lymphocytes (CTLs), which are among the most potent effector cells of the immune system [15]. One strategy to leverage the anti-tumor activity of CTLs involves activating pre-existing, endogenous tumor-directed CTL clones by administering immune checkpoint inhibitors or mAbs that block receptors such as PD-1 and CTLA-4 which are involved in T-cell-inhibitory pathways [16]. The use of immune checkpoint inhibitors like pembrolizumab, nivolumab, and ipilimumab has been remarkably effective for treating many types of solid tumors, but, unfortunately, the activity of these agents is limited in most subtypes of B-NHL [17,18]. Instead, redirecting CTLs using CAR-T or BsAbs has been a much more effective approach. An in-depth discussion of CAR-T is outside the scope of this review, but readers are directed to one of several excellent review articles on the subject [19,20].

As previously mentioned, T-cell-redirecting BsAbs simultaneously bind the T-cell antigen CD3 and a tumor-associated antigen. This effectively brings the immune cell and malignant cell in proximity with one another and creates a cytotoxic synapse that bypasses the interaction between major histocompatibility complex (MHC) proteins and the T-cell receptor (TCR) which are normally required to trigger cytotoxicity [21]. Therefore, BsAbs do not rely on the tumor cell to present an antigen via MHC, which is beneficial when treating tumors like DLBCL which are known to downregulate MHC as a mechanism of immune evasion [22].

While the concept of BsAbs emerged decades ago, the development of this class of agents was delayed until more recent technological advances in protein design and manufacturing enabled more efficient production [23]. Bispecific molecules that are engineered to contain an Fc region are referred to as immunoglobulin G (IgG)-like BsAbs; these resemble native immunoglobulins in their structure and have pharmacokinetic (PK) properties like mAbs. Bispecific molecules that lack an Fc region include bispecific T-cell engagers (BiTEs) and dual-affinity retargeting proteins (DARTs). BiTEs and DARTs are much smaller molecules compared to IgG-like BsAbs, and while this may allow for greater tissue penetration, it also results in rapid renal clearance necessitating frequent dosing intervals or continuous intravenous (IV) infusions to achieve biologically active concentrations.

Blinatumomab, a CD3xCD19 BiTE, was the first T-cell-redirecting molecule developed for B-cell malignancies, and it continues to have an important role in treating patients with B-cell acute lymphoblastic leukemia (B-ALL). However, for R/R DLBCL, while blinatumomab demonstrated reasonable single-agent activity with an overall response rate (ORR) of 55%, logistical challenges associated with its continuous IV administration and a high incidence of neurological toxicity hindered its further development for this disease [24]. Instead, development of IgG-like BsAbs was prioritized in B-NHL, and a discussion of these agents will be the focus of the following sections.

## 3. Structural Features of IgG-like Bispecific Antibodies

The presence of the Fc domain in IgG-like BsAbs prolongs their half-life by reducing the rate of renal excretion and enabling neonatal Fc receptor recycling, a biologic process which delays the catabolism of IgG-like antibodies [25]. However, the Fc receptor can also trigger unwanted effects, including premature T-cell activation and the unintended killing of endogenous T-cells caused by interactions between the Fc domain on the BsAb and the Fc-gamma receptor (FcRy) on other immune effector cells. To prevent this issue, IgG-like BsAbs are engineered to include silencing mutations in the Fc domain that inhibit interactions with FcRy while maintaining binding with the neonatal Fc receptor to retain favorable PK properties [26].

Connected to the Fc domain are two or more fragment antigen-binding (Fab) regions. One of the Fab regions targets CD3, which is a molecule associated with the TCR on endogenous T-cells which transmits activation signals to the cell. Targeting CD3, as opposed to other T-cell antigens, can trigger CD3 to send an activation signal, thereby allowing for T-cell activation without the need for antigen presentation to the TCR [27]. Both CD8+ and CD4+ T-cells express CD3, and both T-cell subsets have been shown to directly participate in the cytolytic activity of the target cells [26]. However, the impact of broadly activating all CD3+ T-cells is poorly understood, and the overactivation of certain T-cell populations could theoretically have undesirable effects, so further research into these interactions is warranted. Additionally, the intensity of TCR pathway signaling, which is related to Fab-binding affinity for CD3, influences whether a T-cell will engage in cytolytic activity, secrete cytokines, and proliferate as opposed to entering an exhausted state and undergoing apoptosis [28,29,30]. The binding interactions between CD3 and BsAbs may have important implications regarding the safety and efficacy of these agents, so studies to further understand these interactions and optimize BsAb structure are of high priority.

The other Fab regions of a BsAb target a tumor-associated antigen. The ideal tumor-associated antigen is an extracellular protein with uniform expression in tumor cells but limited expression in essential healthy tissues to minimize on-target and off-tumor side effects. Fluctuating levels of antigen expression because of antigen loss or downregulation in response to therapy should be minimal. A preclinical study evaluating several different B-cell membrane proteins (CD20, CD22, CD24, CD37, CD70, CD79b, CD138, and HLA-DR) as potential BsAb targets demonstrated wide variation in their ability to trigger cytotoxic activity [26]. CD20 emerged as one of the most potent targets, whereas CD138 and HLA-DR did not generate any cytotoxic activity despite high levels of expression, highlighting the fact that antigen density is not the only factor to consider when selecting a therapeutic target. CD20 has other favorable properties as a target antigen: it is exclusively expressed on B-cells, and despite the widespread use of CD20-targeting therapies for most subtypes of B-NHL, the loss of CD20 expression or mutations involving CD20-binding epitopes are rarely observed in patients who relapse [31,32]. As a result, CD20 is the tumor-associated antigen targeted by all BsAbs currently approved for B-NHL. Of note, mosunetuzumab binds the same epitope as rituximab, whereas glofitamab binds the same epitope as obinutuzumab, and epcoritamab binds the same epitope as ofatumomab. The specific CD20 epitope being targeted by BsAbs is an important consideration when developing combination therapies.

CD19 is another pan-B-cell antigen that is the current target for all commercial CAR-T products used for B-NHL. CD19 is also the target of tafasitamab and loncastuximab tesirine, which are both used in R/R DLBCL [33,34]. Compared to CD20, CD19 expression tends to be more variable in malignant cells, and treatment-related antigen loss is more of a concern, with a loss of CD19 expression reported in up to 30% of patients who relapse after CAR T-cell therapy [35]. While CD19xCD3 IgG-like BsAbs are currently in clinical development for B-NHL, the rate of antigen loss associated with these agents must be carefully monitored and taken into consideration when sequencing therapies.

## 4. Bispecific Antibody Manufacturing

Assembly of IgG-like BsAbs requires combining two different heavy chains and two different light chains in a specific structural orientation. When a single producer cell is programmed to generate two heavy chains and two light chains that are then allowed to randomly pair with one another, the manufacturing process is very inefficient. The use of the “knobs-into-holes” technology is a common strategy to improve BsAb manufacturing efficiency. With this technology, mutations that alter the physical structure of the CH3 domains on the two heavy chains are introduced such that one heavy chain expresses “knobs” that fit into “holes” on the other to facilitate the desired heterodimerization [36]. Similarly, the CrossMab technology induces crossover of the heavy chain and light chain domains within one Fab region, which forces a specific structural orientation when the antibody is being assembled [37]. Such technological advances resulted in a much more efficient manufacturing of BsAbs, which, in turn, allowed for their further clinical development. Additional efforts to improve the design and efficiency of BsAb manufacturing are ongoing [38].

Current BsAb manufacturing platforms can generate antibodies containing more than two Fab regions. For example, glofitamab is a BsAb that has bivalent binding to CD20; that is, it contains two Fab regions that target CD20 while maintaining monovalent (one Fab) binding to CD3. This 2:1 bivalent structure led to increased binding affinity and anti-tumor activity compared to monovalent binding in preclinical studies [39,40]. The spatial orientation of the Fab domains in bivalent BsAbs influences anti-tumor activity. For glofitamab, the “head-to-tail” orientation, which involves using a flexible linker to attach the CD3 Fab with one of the CD20 Fabs on the same side of the antibody structure, results in more potent cytolytic activity compared to when the CD3 Fab region is on one arm of the IgG structure and the two CD20 Fabs are on the other [40]. Multivalent antibodies that target multiple tumor-associated antigens or engage co-stimulatory molecules are also in development.

## 5. Pharmacokinetic Properties

The PK profile of BsAbs shares similarities with conventional mAbs but also depends on various factors including their format, molecular weight, physicochemical properties, interaction with Fc receptors, and binding targets [41].

Like other therapeutic antibodies, BsAbs are poorly absorbed through the gastrointestinal tract and therefore administered via IV or subcutaneous (SC) routes. SC administration generally results in a slower absorption rate and lower peak drug concentrations compared to IV administration, which could mitigate CRS and other adverse events. After SC administration, absorption through the lymphatic system likely occurs at a slow rate, reaching maximum concentrations around three to seven days post dosing [42].

The distribution of BsAbs depends on their molecular construct and binding affinities to both effector and target cells. In general, BsAbs exhibit small volumes of distribution due to their large molecular size. For large BsAb, extravasation from blood vessels to tissue interstitial space primarily occurs via convection [42,43] and FcRn-mediated transcytosis if a functional Fc domain is present [43,44]. In general, therapeutic antibodies have a limited ability to penetrate the blood–brain barrier (BBB) and enter the central nervous system (CNS). However, early evidence suggests that glofitamab can partially penetrate the BBB and induce a clinical response in patients with secondary CNS involvement of DLBCL [45]. A possible future strategy to improve the penetration of BsAbs into the CNS is to engineer a multivalent structure that includes an Fab region that binds one of the BBB transport receptors.

## 6. Bispecific Antibodies in Clinical Practice

The treatment landscape for B-NHL has significantly changed in recent years, with the development of CAR-T representing one of the major advances in the field. For DLBCL, CAR T-cell therapy was initially approved for patients who have received ≥2 lines of prior therapy and subsequently the standard second-line treatment for patients who relapse within 1 year of completing their initial therapy and for those who are unfit for autologous stem cell transplantation [9,46]. CAR T-cell therapy is also commercially available to treat FL after two or more lines of systemic therapy [47,48,49]. The manufacturing period for autologous CAR-T can take several weeks, which is problematic for patients with rapid disease progression. Additionally, centers that administer CAR-T require special accreditation and infrastructure such as cell processing and apheresis capabilities, limiting access to treatment for patients who do not live near large medical centers. BsAbs have lower rates of severe toxicity and are generally easier to administer than CAR-T. Table 1 summarizes some of the key differences between BsAbs and CAR-T.

Three anti-CD20xCD3 BsAbs are currently approved by the FDA to treat B-NHL. Glofitamab and epcoritamab are approved to treat aggressive B-NHL after two or more lines of therapy, whereas mosunetuzumab and epcoritamab are available to treat FL after two or more lines of therapy. Table 2 summarizes the key features of the current FDA-approved BsAbs, including structural characteristics, dosing schedules, and toxicity considerations. The following section highlights clinical data from the pivotal studies that led to the approval of these agents and highlights ongoing clinical trials involving these agents.

### 6.1. Mosunetuzumab

Mosunetuzumab is a fully humanized IgG1 monovalent CD20xCD3 BsAb. A phase 1 trial enrolled patients with indolent and aggressive B-NHL and demonstrated a tolerable safety profile using a step-up dosing schedule, as well as promising efficacy [50]. The ORRs were 66.2% (complete response [CR] 48.5%) and 34.9% (CR 19.4%) among patients with indolent and aggressive B-NHL, respectively. Based on the higher response rate in indolent B-NHL, further development of mosunetuzumab focused on FL. A phase 2 expansion cohort enrolled 90 patients with R/R FL who received two or more prior lines of therapy [10]. Participants received time-limited treatment with mosunetuzumab monotherapy (see Table 2 for the dosing schedule). With 18.3 months of follow-up, the ORR was 80% (CR 60%), and the time to first response was 1.4 months (range 1.2–2.9). The median duration of response (DoR) and progression-free survival (PFS) were 22.8 months and 17.9 months, respectively. Notably, 70% of patients who achieved CR maintained their response for at least 18 months. The 18-month overall survival (OS) rate was 90%. A prespecified subgroup analysis demonstrated similar response rates in high-risk groups, including patients with disease progression with 24 months of initial therapy. Correlatives studies did not reveal an association between baseline CD20 expression by IHC and response, and responses were observed in patients with low CD20 expression (as low as 44%).

CRS occurred in 40 (44%) patients and was predominantly of a low grade. Only one patient had grade 3 CRS. One patient who had circulating tumor cells when treatment was initiated experienced grade 4 CRS. CRS occurred most often after the initial step-up dose on cycle 1 day 1, with a median time to onset of 5 h (range 3–9), or after the first full dose on cycle 1 day 15, with a median time to onset of 27 h (range 8–44). CRS occurred after the cycle 2 day 1 dose in 10.3%, with a median time to onset of 38 h (range 32–57). Only 2.4% of CRS events occurred during cycles 3 and beyond. Neurological adverse events consistent with ICANS occurred in five (5.5%) patients, and all events were grades 1–2. Other common adverse events included fatigue (37%), headache (30%), and rashes (15%).

Based on these results, the FDA granted accelerated approval to mosunetuzumab in December 2022. Of note, a subcutaneous formulation of mosunetuzumab is currently under development [51].

### 6.2. Epcoritamab

Epcoritamab is a monovalent CD20×CD3 BsAb formulated for subcutaneous administration. The phase 1/2 EPCORE NHL-1 study evaluated single-agent epcoritamab in patients with R/R B-NHL and demonstrated favorable safety profile and efficacy in both FL and DLBCL, which led to further development of the drug in both diseases [52].

#### 6.2.1. Epcoritamab for Large-B-Cell Lymphoma

An expansion cohort of the EPCORE NHL-1 study enrolled 157 patients with R/R DLBCL who had received two or more prior therapies [12]. Patients received subcutaneous epcoritamab in 28-day cycles, and treatment was continued until disease progression or intolerance. The median age of the participants was 64 years (range 20–84), 96 (61%) patients had primary refractory disease, and 61 (38.9%) had had prior CAR T-cell therapy. At a median follow-up of 10.7 months, the ORR was 63.1% (CR 38.9%). The median time to response was 1.4 months (range 1.0–8.4). Nine patients converted from PR to CR at or after the 9-month disease assessment. The median DoR was 12.0 months, and an estimated 88.7% of patients who achieved CR maintained their response at 9 months. The median PFS was 4.4 months, and the median OS was not reached. For key subgroups, including patients with primary refractory disease and those who had received prior CAR-T, the response rates were consistent with those of the overall study population. A total of 49 (45.8%) out of 107 evaluable patients achieved undetectable minimal residual disease (MRD), assessed using the ClonoSeq assay, and this was associated with an improved PFS.

CRS occurred in 49% of patients and was predominantly grade 1 or 2 in severity. Grade 3 CRS events occurred in four patients, and there were no grade 4 or 5 events. Most CRS events occurred after the administration of the first full dose of epcoritamab on cycle 1 day 15, with a median time to onset of 20 h after the dose. CRS events were rare in cycles 2 and beyond. ICANS occurred in ten (6.4%) patients, including seven patients with grade 1 events, two patients with grade 2 events, and one patient with a grade 5 event. Treatment-emergent grade 3–4 infections occurred in 23 (14.6%) patients. Other adverse events included neutropenia (21.7%), injection site reaction (19.7%), and headache (14%).

In May 2023, the FDA granted accelerated approval to epcoritamab for R/R DLBCL after two or more prior therapies. Inpatient observation for 24 h after administering the first full dose on cycle 1 day 15 is required as per the US package insert, although efforts are underway to safely transition this completely to the outpatient setting (NCT05451810).

#### 6.2.2. Epcoritamab for Follicular Lymphoma

A second expansion cohort of EPCORE NHL-1 enrolled 128 patients with R/R FL who had received two or more lines of systemic therapy [13]. The participants received subcutaneous epcoritamab in 28-day cycles until disease progression or intolerance. Notably, this study also included a dose optimization cohort that incorporated an additional intermediate step-up dose to further reduce rates of CRS and ICANS. At a median follow-up of 17.4 months, the ORR was 82% (CR 62.5%). An estimated 58.4% of all responding patients and 72.2% of patients in CR maintained their response at 18 months. Subgroup analysis identified refractory disease and the receipt of ≥ 4 lines of therapy as factors associated with an inferior response.

The rate of grade 1–2 CRS was 65%, whereas grade 3 CRS occurred in only 2% of patients. In the pivotal cohort, most CRS events occurred after administration of the first full dose on cycle 1 day 15, with a median time to onset of 15 h after dose administration. ICANS was reported in 6% of patients, with all events being of grade 1 or 2 in severity. Among the 86 patients who received the additional intermediate step-up dose in the dose optimization cohort, the CRS rate was low (40% grade 1, 9% grade 2, and 0% ≥ grade 3), and no episodes of ICANS were observed.

The FDA granted accelerated approval to epcoritamab for patients with R/R FL in June 2024. Based on the results of the dose optimization cohort, inpatient monitoring for FL is not required when an extra intermediate dose is incorporated into the step-up schedule.

### 6.3. Glofitamab

Glofitamab is a bivalent CD20xCD3 BsAb consisting of two anti-CD20 Fab regions and one anti-CD3 Fab region oriented in a head-to-tail configuration. Administering obinutuzumab, an anti-CD20 mAb, to deplete B-cells prior to the initial dose of glofitamab reduced T-cell activation and cytokine secretion in preclinical experiments, so this approach was utilized in subsequent clinical studies as a CRS mitigation strategy [40].

A phase 1 study of glofitamab in R/R DLBCL utilized obinutuzumab pre-treatment and established the step-up dosing schedule during cycle 1 [53]. The phase 2 dose expansion cohort enrolled patients with R/R DLBCL who had received two or more lines of therapy [11]. Glofitamab was administered intravenously in 21-day cycles for up to 12 cycles. The patients received pre-treatment with 1000 mg of obinutuzumab on cycle 1 day 1 and then started the glofitamab ramp-up 1 week later (see Table 1 for the dosing schedule). The study enrolled 154 patients, of whom 90 (58%) had primary refractory disease and 55 (33%) had received prior CAR-T. With a median follow-up of 12.6 months, the ORR was 52% (CR 39%), and the median time to CR was 1.4 months, corresponding to the first response assessment in the study. Seventy-eight percent of patients who achieved CR maintained this response at 12 months. The estimated 12-month PFS and OS rates were 37% and 50%, respectively. A prespecified subgroup analysis demonstrated that the CR rates were consistent among patients who had received prior CAR T-cell and those who had not, as well as for patients older and younger than the age of 65 years.

CRS occurred in 97 (63%) patients and was primarily of a low grade. Grade 3 and 4 CRS occurred in 3% and 1% of patients, respectively. Seven patients required admission to the intensive care unit for the management of CRS. Neurological events consistent with ICANS developed in 12 (8%) patients, with events of grade 3 or higher occurring in 3% of patients.

Based on these phase 2 data, the FDA granted accelerated approval of glofitamab for the treatment of R/R DLBCL after two or more lines of systemic therapy. Admission for inpatient observation after the initial 2.5 mg dose of glofitamab is required as per the US package insert.

Of note, glofitamab is now the first BsAb to demonstrate an overall survival benefit in a randomized phase 3 study. The STARGLO study randomized patients with R/R DLBCL who were ineligible for ASCT according to a 2:1 design to receive either fixed-duration glofitamab in combination with gemcitabine and oxaliplatin (glofit-GemOx) or rituximab plus GemOx (R-GemOx). With a median follow-up of 20.7 months, the median OS was 25.5 months for the patients receiving glofit-GemOx compared to 12.5 months for those receiving R-GemOx, translating to a hazard ratio of 0.62 (95% CI, 0.42–0.88, and *p* = 0.006). The magnitude of improvement with glofit-GemOx was substantial, and this was in a patient population that was ostensibly not eligible for CAR-T. While these results do not change the current standard of care for patients eligible for CAR-T, they raise some important questions about treatment sequencing for patient with R/R DLBCL. The results of phase 3 studies combining BsAbs with CIT for the first-line therapy of DLBCL, discussed in more detail below, are eagerly awaited [54].

### 6.4. Odronextamab

Odronextamab is a CD20xCD3 BsAb that showed significant activity in a phase 1 study [55]. The phase 2 ELM-2 study included cohorts with R/R DLBCL and R/R FL. Among 127 patients with R/R DLBCL, the ORR was 52% (CR 31.5%), and the median DOR was 10.2 months [56]. Among the 85 efficacy-evaluable patients treated in the R/R FL cohort, the ORR was 81% (CR 75%), and the median DOR was 18.2 months [57]. Despite these results, odronextamab has not been approved by any regulatory authority and is not commercially available. The initial biologic license application to th0e FDA was declined, citing the enrollment status of confirmatory studies evaluating this agent as opposed to any safety, efficacy, or manufacturing concerns.

Currently, odronextamab is being investigated in combination therapies in phase 3 trials to further evaluate its efficacy and safety in earlier lines of therapy. In DLBCL, the OLYMPIA-3 trial (NCT06091865) is evaluating odronextamab with CHOP compared to R-CHOP in previously untreated patients, and OLYMPIA-4 includes R/R DLBCL. In FL, it is also being evaluated in the phase 3 OLYMPIA-2 (NCT06097364) trial, comparing odronextamab plus chemotherapy vs. rituximab plus chemotherapy.

In summary, BsAbs such as mosunetuzumab, epcoritamab, and glofitamab represent advancement in the treatment of patients with R/R B-NHL. These agents offer several advantages over traditional therapies, including immediate availability, a favorable safety profile, and broader accessibility compared to CAR-T therapy. Ongoing clinical trials are exploring BsAbs-based combination therapies to improve efficacy and optimize treatment algorithms in B-NHL.

### 6.5. Efficacy of Bispecific Antibodies After CAR-T

The optimal sequencing of BsAbs and CAR-T remains to be defined. In the DLBCL expansion of EPCORE NHL-1, the 69 (39%) patients treated with epcoritamab after prior CAR-T had a numerically, but not statistically significant, lower ORR (54% vs. 69%) and CR rates (34% vs. 42%) compared to patients who had not received CAR-T [12]. The 15 patients who were not refractory to prior CAR-T had improved response rates, with an ORR 80% (CR 53%). Among the 51 (33%) patients treated with glofitamab after prior CAR-T, the ORR was 42% (CR 35%); the CR rate did not significantly differ from that of patients who did not receive prior CAR-T.

Real-world data that address this issue are beginning to emerge and show some conflicting results. A retrospective analysis of 387 R/R DLBCL patients in Spain and the UK who progressed after CAR-T reported an ORR of 51% (CR 36%) among patients who received a BsAb as the next line of therapy, which was significantly better than other therapies evaluated which included immune checkpoint inhibitors, lenalidomide-based therapy, and CIT [58]. The estimated 12-month PFS and OS for patients receiving post-CAR-T BsAbs were 32% and 50%, respectively. In contrast to this report, an analysis of the French DESCAR-T registry showed that, In contrast to this report, an analysis of the French DESCAR-T registry showed that, among the 238 patients who had relapsed after CAR-T, 11 of the 154 (7%) patients who had received subsequent therapy were treated with CD20xCD3 BsAB. These patients had an ORR of only 14% and a median PFS of 3.7 months, which were not significantly different from other treatment strategies, which were not significantly different from other treatment strategies [59]. Lastly, a large cohort of US patients reported that, among 10 patients who received BsAb after the failure of CAR-T, the ORR was 50% (CR 20%) [60].

Additional data with a longer follow-up are required to comment on the relative efficacy of BsAbs before and after CAR-T, but, for now, it appears that using BsAbs to treat patients relapsing after CAR-T is a reasonable treatment strategy. As BsAbs are evaluated in earlier lines of therapy, attention to the impact BsAbs have on the efficacy of subsequent CAR-T is needed; early reports suggest no detrimental impact on efficacy [61], but there are currently limited data on the subject, and there is a theoretical risk that BsAb exposure could induce T-cell exhaustion and impact CAR-T efficacy, so ongoing attention to this issue is required.

### 6.6. Ongoing Studies on Bispecific Antibodies in B-NHL

The promising single-agent activity of BsAbs in R/R B-NHL inspired numerous trials investigating their use in combination with other agents, in earlier lines of therapy, and in other B-NHL subtypes. Table 3 summarizes ongoing clinical trials involving CD20xCD3 BsAbs.

#### 6.6.1. DLBCL and Other Aggressive B-NHL

Clinical trials investigating BsAbs as part of first-line therapy for DLBCL are actively enrolling patients. Phase 2 studies looking at epcoritamab and glofitamab in combination with cyclophosphamide, doxorubicin, vincristine, and prednisone (CHOP)-based chemotherapy with or without rituximab demonstrated promising response rates and led to the development of phase 3 studies. A phase 3 study investigating polatuzumab, rituximab, cyclophosphamide, doxorubicin, and prednisone (Pola-R-CHP) with or without glofitamab (NCT06047080) and another study of R-CHOP with or without epcoritamab (NCT05578976) are actively recruiting patients. BsAbs are also being studied as a first-line therapy for older and frail adults who are ineligible for full-dose chemotherapy or anthracyclines (NCT05660967).

For R/R DLBCL, the combination of BsAbs with conventional salvage chemotherapy or other targeted agents prior to planned consolidation with ASCT or CAR-T is an area with multiple ongoing phase 2 studies. Consolidation therapy with BsAbs following CAR T-cell therapy is another approach that is being investigated. Lastly, there are several studies evaluating BsAb combinations in patients who are ineligible for or have relapsed after ASCT and CAR-T that are actively recruiting participants.

#### 6.6.2. Follicular Lymphoma

BsAbs are also being evaluated as a first-line therapy in FL. An upcoming cooperative group study will be comparing mosunetuzumab to rituximab in patients with untreated follicular lymphoma with a low tumor burden (NCT06337318). For previously untreated FL with a high tumor burden, the EPCORE FL-2 study compares lenalidomide and rituximab (R2) plus epcoritamab to R2. For R/R FL, the phase 3 CELESTIMO study comparing lenalidomide and mosunetuzumab to R2 (NCT04712097) has completed accrual, awaiting the results from the primary analysis.

#### 6.6.3. Other B-NHL Subtypes

While not currently approved by the FDA, BsAbs are being actively investigated in other subtypes of B-NHL. Recruitment is ongoing for a phase 3 study comparing single-agent glofitamab to the investigator’s choice of therapy for R/R mantle cell lymphoma (MCL). Single-arm studies are also investigating BsAbs in combination with Bruton’s tyrosine kinase inhibitors. For R/R marginal-zone lymphoma (MZL), a phase 3 study comparing lenalidomide and mosunetuzumab to the investigator’s choice of therapy is currently recruiting in Europe. Lastly, EPCORE CLL-1 is investigating epcoritamab combinations in patients with R/R chronic lymphocytic leukemia/small lymphocytic lymphoma (CLL/SLL) and Richter’s syndrome (NCT04623541).

Targeted small-molecule inhibitors, such as Bruton’s tyrosine kinase (BTK) inhibitors, are also being explored in combination with bispecific antibodies. The rationale for these combinations stems from the ability of BTK inhibitors to disrupt signaling pathways critical to B-cell survival, potentially sensitizing lymphoma cells to bispecific antibody-mediated T-cell killing. A phase Ib/II trial (NCT04234061) is evaluating epcoritamab in combination with the BTK inhibitor ibrutinib in patients with R/R mantle cell lymphoma (MCL) and chronic lymphocytic leukemia (CLL).

## 7. Safety Considerations

BsAbs are generally associated with lower rates of severe side effects compared to CAR-T. As a result, BsAbs should be more feasible to administer in community oncology practices and cancer centers with fewer resources, thus expanding access to care. However, life-threatening side effects can occur during BsAb therapy, so it is critical that all healthcare providers involved in the care of these patients, including emergency department staff, hospitalists, and nurses, know the toxicities and have a management pathway. Institutions that administer BsAbs must develop protocols to help providers triage patients to the appropriate level of care and guide the management of these toxicities when they occur. The following section discusses the major toxicities associated with CD20xCD3 BsABs.

### 7.1. Cytokine Release Syndrome

Treatment with T-cell-engaging BsAbs results in T-cell activation and the release of pro-inflammatory cytokines such as IL-2, IL-6, INF-g, and TNF-a, which results in the clinical manifestation of CRS: fever, hypotension, and hypoxemia [52,62]. CRS is generally less frequent and severe compared to CAR-T, and episodes occur during cycles 1 and 2. Utilizing step-up dosing during cycle 1 reduces the frequency and severity of CRS [63]. Subcutaneous administration appears to be associated with lower rates of CRS compared to IV [51]. Adherence to the prescribing information available for each product regarding the use of premedication and inpatient monitoring is recommended. For patients who develop CRS, its severity is graded using validated scales such as the Lee Criteria published by the American Society of Transplantation and Cellular Therapy [64]. Consensus recommendations for the management CRS with CD20xCD3 BsAbs have been published [65]. In general, the management of CRS involves providing supportive measures like antipyretic and IV fluids, repeating doses of corticosteroids in patients with persistent fevers or grade ≥ 2 CRS, and considering the administration of anti-cytokine therapy such as tocilizumab in refractory or high-grade cases.

### 7.2. Neurological Toxicity

Patients receiving BsAbs may experience adverse events like dizziness or headache during BsAb therapy, although neurological symptoms consistent with immune effector cell-associated neurotoxicity syndrome (ICANS) have also been reported. The pathophysiology of ICANS remains poorly understood, although inflammatory cytokines, disruption of the blood–brain barrier, and infiltration of T-cells and myeloid cells into the CNS have all been proposed as contributing factors [66]. Clinical manifestations of ICANS vary widely and can include tremor, dysgraphia, inattention, confusion, agitation, lethargy, somnolence, seizure, and cerebral edema. Fortunately, ICANS following CD20xCD3 BsAb therapy is less common and severe compared to CD19-directed CAR-T. However, these events can still occur, so patients and their caregivers much be educated on the early signs and symptoms of ICANS so that they can be promptly evaluated. Grading and management of ICANS should follow the same guidelines published for patients who receive CAR-T [64,67].

### 7.3. Infection

Several factors contribute to an increased risk of infection in patients treated with BsAB therapy. Lingering immunosuppressive effects of prior therapies contribute, especially in patients with R/R disease or who have recently received CAR T-cell therapy. BsAbs targeting B-cell antigens like CD19 and CD20 deplete non-malignant B-cells and result in hypogammaglobulinemia. T-cell exhaustion due to prolonged BsAb exposure may further increase the risk of infection [68]. Opportunistic infections, including pneumocystis jirovecii pneumonia, toxoplasmosis, and invasive aspergillosis have been reported. The reactivation of hepatitis B, CMV, and EBV may occur. Patients are more susceptible to viral infections such as influenza, parvovirus, adenovirus, and COVID-19. Cases of progressive multifocal leukoencephalopathy have been reported [69]. Patients being treated with BsAbs must be monitored closely for any signs and symptoms of infection, and treating physicians must be aware of the risk of opportunistic infections. Patients who are actively undergoing treatment likely have an inadequate response to vaccinations. Prophylaxis for herpes virus infections is recommended in all patients, and pneumocystis prophylaxis should also be strongly considered. Immunoglobulin levels should be monitored during therapy, and hypogammaglobulinemic patients with recurrent infections can be supported with intravenous immunoglobulin.

## 8. Mechanisms of Resistance

One proposed mechanism of resistance to BsAbs includes tumor-associated factors such as antigen loss and the presence of certain molecular features in the target cells. An analysis of paired pre-treatment and at-progression tumor samples obtained from patients treated with mosunetuzumab demonstrated that the loss of CD20 expression occurred in 11 out of 32 (34%) patients at the time of progression [70]. Correlative studies from the phase 1 study of glofitamab in R/R B-NHL identified tumor-specific gene expression signatures, including the overexpression of MYC and the downregulation of TP53 targets, which were associated with a poor response to therapy [62]. *MYC* amplification and *TP53* mutation are known molecular abnormalities in B-NHL that can account for these gene expression abnormalities. This study highlights the feasibility of identifying distinct genetic and molecular lymphoma subtypes that are resistant to BsAb therapy. While the mechanism remains unclear, emerging evidence suggests that *TP53* mutations contribute to an immunosuppressive microenvironment and promote immune escape through a variety of downstream effects [71,72,73].

Host factors like immune effector cell fitness have also been implicated in BsAb resistance. For example, patients who progressed on glofitamab in a phase 1 study had T-cells that exhibited a high PD1 signature phenotype prior to therapy, a feature which has been associated with dysfunctional tumor-infiltrating lymphocytes [62]. Additionally, chronic T-cell stimulation from continuous BsAb therapy has been shown to induce T-cell exhaustion and the loss of effector functions [53,68,74]. Exposure to multiple lines of lymphotoxic therapy in patients with B-NHL can impair baseline T-cell fitness and potentially impact the efficacy of BsAbs, and several trials are underway, as described above, to move these agents to earlier lines of therapy, which would mitigate the effects of prior therapies on the host’s immune function.

Our understanding of resistance mechanisms to BsAbs remains limited, so studies exploring this topic are urgently needed to refine patient selection for BsAb and develop strategies to overcome these issues.

## 9. Future Perspectives

The next generation of T-cell-engaging antibodies is already under development. Optimizing structural characteristics like the Fab-binding affinity may improve the efficacy and reduce the toxicity of newer agents. Similarly, designing multi-specific antibodies that target multiple tumor-associated antigens or immune effector cells may improve the activity of this class of agents. Identifying new targets for BsAbs to expand their use to other types of lymphoma and solid tumors is also an area of active investigation.

AZD0486 is a CD19xCD3 BsAb that incorporates a low-affinity CD3-binding moiety to reduce cytokine secretion and toxicity. In a phase 1 study enrolling patients with R/R CD19+ B-NHL, treatment with AZD0486 using a two-dose step-up regimen resulted in an incidence of grade 1–2 CRS of only 22% and no grade ≥3 events observed. The incidence of ICANS was 5.5% with all events being of grade 1 or 2 in severity. AZD0486 demonstrated promising efficacy, especially among patients with R/R FL, where the ORR was 88% (CR 83%), and the median DoR was not reached [75]. GB261 is a CD20xCD3 BsAb under early clinical development that similarly utilizes de-tuned CD3 binding to reduce toxicity [76].

Utilizing multi-specific antibodies to target two or more tumor-associated antigens in addition to CD3 can minimize the effect of tumor antigen loss as a resistance mechanism and increase cytotoxic activity against target cells that have low levels of antigen density. Additionally, tri-specific antibodies that simultaneously target a tumor-associated antigen, CD3, and a co-stimulatory molecule on effector cells, such as CD2, CD28, and 4-1BB, have been shown to increase the anti-tumor activity of effector T-cells and may help decrease T-cell exhaustion during therapy [77,78,79,80,81]. Another strategy that is being evaluated involves the coadministration of glofitamab and RG6333, a CD19xCD28 BsAb which provides a second co-stimulatory signal to effector T-cells via CD28 binding. RG6333 has been shown to enhance the potency of glofitamab in preclinical studies [82], and there is an ongoing phase 1 clinical trial evaluating this combination (NCT05219513).

BsAbs can also be used to leverage the anti-tumor activity of other immune effector cells. AFM13 is a bispecific NK cell engager that simultaneously targets CD30 on tumor cells and CD16 on NK cells [83]. The coadministration of AFM13 and cord blood-derived NK cells demonstrated an ORR of 93% (CR 66%) in patients with heavily pre-treated Hodgkin lymphoma and NHL. The duration of the response was short, but extended dosing schedules and other consolidation strategies are being evaluated [84]. The combination of AFM13, NK cell product AB-101, and low-dose IL-2 is currently being evaluated in a clinical trial for patients with Hodgkin lymphoma and CD30+ peripheral T-cell lymphoma (NCT05883449).

Lastly, there are ongoing efforts to expand the therapeutic potential of T-cell-engaging antibodies to other subtypes of lymphoma. AZD7899 is a BsAb-targeting PD-L1, and TIM-3, a protein associated with resistance to anti-PD-1/PD-L1 therapies in classical Hodgkin lymphoma, is in early clinical development [85]. ONO-4685 is a PD-L1xCD3 BsAb being evaluated in T-cell lymphoma (NCT05079282).

## 10. Conclusions

Currently approved BsAbs have demonstrated impressive efficacy and safety in R/R B-NHL, suggesting their potential to reshape the entire treatment landscape for this malignancy. Some patients with R/R B-NHL achieve deep and durable remission following treatment with current CD20xCD3 BsAbs. However, a longer follow-up is needed to determine whether these agents can cure patients on their own. Identifying predictors of response is necessary, as many patients have suboptimal outcomes with BsAb monotherapy. BsAb-containing combination regimens are under development, and we are beginning to see encouraging phase 3 data emerge in this area. The integration of BsAbs into combination regimens holds great promise for improving patient outcomes and expanding access to cutting-edge therapies. However, much work still needs to be carried out to determine the optimal therapeutic partners for BsAbs and the line of therapy in which they would be best used. Mechanisms of resistance and factors driving the toxicities seen with BsAbs require further characterization so that rational design changes can be incorporated into next-generation products. While many challenges remain, BsAbs provide new hope for long-term remission and improved quality of life for patients with B-NHL.

## Figures and Tables

**Table 1:** Comparison of bispecific antibodies and CAR-T in large-B-cell lymphoma.

	CD20xCD3 Bispecific Antibody	CD19-Directed CAR-T
FDA-Approved Agents	Glofitamab, epcoritamab	Axi-cel, liso-cel, tisa-cel
Current indication	≥2 prior lines of therapy	Second-line therapy: if relapse < 12 months from initial therapy or ASCT ineligible (only axi-cel, liso-cel) ≥2 prior lines of therapy
Availability	Immediately available No risk of manufacturing failure	Delay of 3–5 weeks for manufacturing Manufacturing failure or out-of-specification product possible
Infrastructure and logistical concerns	No special accreditation required No apheresis or cell processing facilities	Facility must be FACT-accredited; Apheresis and cell processing facilities and personnel required; Financial navigators and nurse navigators/transplant coordinators often required
Dosing schedule	Repeated dosing required	One-time administration
CRS rate	All grades: 50–63% Grade ≥ 3: 1–4%	All grades: 42–93% Grade ≥ 3: 2–22%
ICANS rate	All grades: 3–8% Grade ≥ 3: 1–3%	All grades: 30–64% Grade ≥ 3: 10–28%
Grade ≥ infection	15–23% Pneumocystis, CMV reactivation, and PML have been reported	12–19% Wide variety of opportunistic infections reported
Cytopenias	Grade ≥ 3 neutropenia observed in 26–38% receiving BsAb monotherapy	Post-CAR-T cytopenias very common; persistent cytopenias lasting > 9 days reported in 2–45% of patients

CAR-T, chimeric antigen receptor T-cell therapy; axi-cel, axicabtagene ciloleucel; liso-cel, lisocabtagene maraleucel; tisa-cel, tisagenlecleucel; ASCT, autologous stem cell transplant; FACT, Foundation for the Accreditation of Cellular Therapy; CRS, cytokine release syndrome; ICANS, immune effector cell-associated neurotoxicity syndrome; CMV, cytomegalovirus; PML, progressive multifocal leukoencephalopathy; and BsAb, bispecific antibody.

**Table 2:** Properties of FDA-approved CD20xCD3 bispecific antibodies in B-cell non-Hodgkin lymphoma.

		Mosunetuzumab	Epcoritamab		Glofitamab
Structural features		IgG-like 1:1 CD20:CD3 ratio Binds same CD20 epitope as rituximab	IgG-like 1:1 CD20:CD3 ratio Binds same CD20 epitope as ofatumumab		Bivalent, IgG-like 2:1 CD20:CD3 binding ratio Binds same CD20 epitope as obinutuzumab
Route of administration		IV (SC in development)	SC		IV
Terminal half-life		16.1 days	22 days		7.6 days
Cycle length		21 days	28 days		21 days
Treatment schedule and duration		Dose administered every 21 days after completing C1 step-up Duration: treat for 8 cycles if in CR, 17 cycles if PR/SD	Doses administered: C1–3: days 1,8, 15, and 22 C4–9: days 1 and 15 C10+: day 1 Duration: treat until progression Dose optimization for patients with FL: additional 3 mg intermediate step-up dose added to C1D15		Dose administered every 21 days after C1 step-up Treat for 12 cycles C1D1 obinutuzumab pre-treatment to mitigate CRS
Indications		FL	DLBCL	FL (dose optimization)	DLBCL
Dosing		C1 D1: 1 mg C1 D8: 2 mg C1 D15: 60 mg C2 D1: 60 mg Cycle 3+ Day 1: 30 mg	C1D1: 0.16 mg C1D8: 0.8 mg C1D15: 48 mg C1D22: 48 mg C2+: 48 mg	C1D1: 0.16 mg C1D8: 0.8 mg C1D15: 3 mg C1D22: 48 mg C2+: 48 mg	C1D1: obinutuzumab 1000 mg C1D8: 2.5 mg C1D15: 10 mg C2-C12D1: 30 mg
Hospitalization during step-up		Not required	24 h after C1D15 dose	Not required with dose optimization	24 h after C1D8 dose
CRS incidence	Grade 1	26%	32%	40%	47%
	Grade 2	17%	15%	9%	12%
	Grade 3	1%	3%	0%	3%
	Grade 4	1%	0%	0%	1%
	Grade 5	0%	0%	0%	0%
Timing of CRS events (median time to onset, when reported)		C1D1: 23.3% (5 h) C1D8: 5.6% (20 h) C1D15: 36.4% (27 h) C2D1: 10.2% (38 h) C3+: 2.4%	C1D1: 5.8% C1D8: 11.8% C1D15: 42.8% (20 h) C1D22: 4.9% C2+: 3%	C1D1: 11% C1D8: 6% C1D15: 14% C1D22: 36% (60 h) C2+ 10%	C1D8: 42.8% (13.5 h, range 6.5–52) C1D15: 25.2% C2D1: 26% C3+D1: 0.9%
ICANS incidence	Grade 1–2	3%	5.8%	0%	5%
	Grade 3	0%	0%	0%	3%
	Grade 4	0%	0%	0%	
	Grade 5	0%	0.6%	0%	0%

IV, intravenous; SC, subcutaneous; C, cycle; D, day; CR, complete response; PR, partial response; SD, stable disease; DLBCL, diffuse large-B-cell lymphoma; FL, follicular lymphoma; CRS, cytokine release syndrome; and ICANS, immune effector cell-associated neurotoxicity syndrome.

**Table 3:** Key clinical trials involving CD20xCD3 bispecific antibodies in B-cell non-Hodgkin lymphoma.

Clinical Setting	Trial Name/NCT	Treatment Regimen	Phase	Key Eligibility/Design Features
**Diffuse Large-B-cell Lymphoma**				
First-line treatment	NCT06047080 SKYGLO	Glofitamab + R-pola-CHP vs. R-pola-CHP	III	IPI 2–5
	NCT05578976 EPCORE DLBCL-2	Epcoritamab + R-CHOP vs. R-CHOP	III	IPI 2–5
	NCT06091865 OLYMPIA-3	Odronextamab + CHOP vs. R-CHOP	III	IPI 2–5
	NCT06045247	Epcoritamab + R-mini-CVP	II	Older unfit/frail and anthracycline ineligible
Salvage therapy prior to transplant/cell therapy	NCT05852717	Epcoritamab + R-GDP	II	After cycle 3, patients may proceed to ASCT, CAR-T, or epcoritamab monotherapy at the investigator’s discretion
Peri-CAR-T	NCT05633615 SWOG 2114	Mosunetuzumab vs. polatuzumab vs. mosunetuzumab + polatuzumab vs. observation	II	Patients with PR or SD on day +30 PET scan after CAR-T are randomized to an experimental arm
	NCT06071871 PORTAL	Glofitamab + polatuzumab	II	Evaluating regimen as bridging therapy for CAR-T; patients not in CR on day +30 resume treatment
	NCT06238648	Epcoritamab vs. Observation	II	Patients randomized if not in CR after CD19-directed CAR-T
	NCT06213311	Glofitamab + Axi-cel	II	Second-line therapy
Other combinations for R/R disease	NCT05283720 M22-132	Epcoritamab + golcadamide	II	Part of a basket study evaluating epcoritamab-containing regimens
	NCT04970901 LOTIS-7	Loncastuximab + glofitamab or mosunetuzumab	Ib	Part of a basket study evaluating loncastuximab-containing regimens
	NCT05171647 SUNMO	Mosunetuzumab + polatuzumab vs. R-GemOx	III	Subcutaneous mosunetuzumab used
**Follicular Lymphoma**				
First-line therapy	NCT06337318 S2308	Mosunetuzumab vs. Rituximab	III	Low tumor burden
	NCT06097364 OLYMPIA-2	Odronextamab + CHOP/CVP vs. R-CHOP/CVP	III	High tumor burden
	NCT06284122 MorningLyte	Mosunetuzumab + lenalidomide vs. R^2^	III	High tumor burden, FLIPI2-5 eligible
R/R FL	NCT05409066 EPCORE FL-1	Epcoritamab + R^2^ vs. R^2^	III	Time-limited therapy (12 cycles)
	NCT04712097 CELESTIMO	Mosunetuzumab + lenalidomide vs. R^2^	III	Time-limited therapy (12 cycles)
**Marginal Zone Lymphoma**				
First-line therapy	NCT04792502	Mosunetuzumab + lenalidomide	II	Mosunetuzumab monotherapy for 4 cycles then response-adapted treatment with len; also has an FL cohort
	NCT05783596	Glofitamab + obinutuzumab	II	High tumor burden; also has an FL cohort
R/R MZL	NCT06006117	Mosunetuzumab + lenalidomide vs. R-chemo	III	Eligibility: extra-nodal, nodal, and splenic MZL with 1–3 prior lines of systemic therapy
**Mantle cell lymphoma**				
First-line therapy	NCT05861050	Glofitamab + obinutuzumab + venetoclax + lenalidomide	I/II	Enrolling high-risk patients
R/R MCL	NCT06084936 GLOBRYTE	Glofitamab vs. Investigator’s choice	III	Prior covalent BTKi exposure
	NCT05833763 GOLDILOX	Glofitamab + pirtobrutinib	II	Prior covalent BTKi exposure
	NCT06192888	Glofitamab + lenalidomide	I	Prior BTKi exposure
	NCT06054776	Glofitamab + obinutuzumab + acalabrutinib	II	
	NCT05283720	Epcoritamab + ibrutinib; Epcoritamab + ibrutinib + venetoclax; Epcoritamab + pirtobrutinib	II	Basket study of epcoritamab combinations in NHL

R-pola-CHP—rituximab, polatuzumab, cyclophosphamide, doxorubicin, and prednisone; R-CHOP—rituximab, cyclophosphamide, doxorubicin, vincristine, and prednisone; IPI—international prognostic index; CVP—cyclophosphamide, vincristine, and prednisone; R-GDP—rituximab, gemcitabine, dexamethasone, and cisplatin; CAR-T—chimeric antigen receptor T-cell therapy; ASCT—autologous stem cell transplant; CR—complete response; PR—partial response; SD—stable disease; Axi-cel—axicabtagene ciloleucel; R-GemOx—rituximab, gemcitabine, and oxaliplatin; R^2^—lenalidomide plus rituximab; FLIPI—follicular lymphoma international prognostic index; and BTKi—Bruton’s tyrosine kinase inhibitor.

## Data Availability

No new data were created or analyzed in this study. Data sharing is not applicable to this article.
